# Underdiagnosis and Ethnic Differences in Community‐Based Brain Health Screening

**DOI:** 10.1002/alz70857_103624

**Published:** 2025-12-25

**Authors:** Rafi Haddad, Rachel Ben‐Hayun, Andjelika Milicic, Rawan Ayoub, Yarovinsky Natalya, Tali Fisher, Judith Aharon‐Peretz, David Tanne

**Affiliations:** ^1^ Global Brain Health Institute, University of California, San Francisco, San Francisco, CA, USA; ^2^ Rambam Health Care Campus, Haifa, Israel; ^3^ Technion‐Israel Institute of Technology, Haifa, Israel; ^4^ University of California San Francisco, San Francisco, CA, USA

## Abstract

**Background:**

In Israel, Arabs experience higher mortality rates from cerebrovascular disease and greater prevalence of vascular cognitive impairment compared to Jews. This study evaluated cognitive disparities between community‐dwelling Arabs and Jews with comparable cerebrovascular risk factors (CVRF).

**Method:**

We enrolled 115 participants (age: 67.07 ± 5.95; education: 15.28 ± 4.16), including 59 Arabs (age: 64.17 ± 5.38; education: 14.76 ± 5.07) and 56 Jews (age: 70.16 ± 4.91; education: 15.82 ± 2.84), all withouxt known cognitive impairment but with at least one CVRF. Participants completed a cognitive symptoms questionnaire in their primary language and underwent the Montreal Cognitive Assessment (MoCA) and the Tablet‐based Cognitive Assessment Tool (TabCAT) Brain Health Assessment (BHA) battery, which included Birdwatch (visial associative memory), Match (executive function and processing speed), and Line Orientation (visuospatial skills) tasks. Clinical characteristics were analyzed using Student's t‐test, Mann‐Whitney U test, and Chi‐squared tests as appropriate. Participants reported the number of days they engaged in ≥30 minutes of physical activity in the prior week.

**Results:**

Cognitive symptoms were reported by 41 participants (35%), including 28 Arabs (68%) and 13 Jews (32%) (*p* = 0.007). Arabs were younger than Jews (*p* < 0.001), with no significant differences in sex or education between groups (*p* = 0.45 and *p* =  0.17, respectively). Participants with and without cognitive symptoms were similar in age, sex, and education (*p* = 0.73, *p* =  0.90, and *p* =  0.30, respectively). Those with cognitive complaints scored lower on the Match task (*p* = 0.002) but not on Birdwatch (*p* = 0.32), Line Orientation (*p* = 0.18), or MoCA (*p* = 0.27). CVRF prevalence, including hypertension, diabetes, hyperlipidemia, and smoking, was similar between participants with and without cognitive symptoms. Participants without cognitive symptoms reported more physical activity days in the previous week (*p* = 0.01).

**Conclusion:**

In our community‐based health screening, the majority of individuals reporting cognitive symptoms were Arabs. Participants with cognitive symptoms exhibited poorer performance on an executive function task, suggesting the possibility of underdiagnosed cognitive disorders. Additionally, greater physical activity was associated with fewer cognitive symptoms, emphasizing the crucial role of exercise in supporting brain health.

